# Associations between Depression, Depressive Symptoms, and Incidence of Dementia in Latin America: A 10/66 Dementia Research Group Study

**DOI:** 10.3233/JAD-190148

**Published:** 2019-05-21

**Authors:** Lena Johansson, Mariella Guerra, Martin Prince, Helena Hörder, Hanna Falk, Brendon Stubbs, A. Matthew Prina

**Affiliations:** a Department of Psychiatry and Neurochemistry, Sahlgrenska Academy, Centre for Ageing and Health (AgeCap) at the University of Gothenburg, Sweden; b Institute of Memory, Depression and Disease Risk, Lima, Peru; c Department of Health Service and Population Research, Institute of Psychiatry, Psychology and Neuroscience, King’s College London, London, United Kingdom; d Department of Physiotherapy, South London and Maudsley NHS Foundation Trust, London, United Kingdom

**Keywords:** Dementia, depression, developing countries, epidemiology, risk factors

## Abstract

**Background:**

A growing body of evidence suggests that depression is related to dementia in older adults. Previous research has been done in high-income countries and there is a lack of studies in low- and middle income countries (LMICs).

**Objective:**

To examine the relationship between depressive symptoms and incidence of dementia in a population-based study of older adults in Latin America.

**Methods:**

The study is a part of the 10/66 Dementia Research Group’s population survey and includes 11,472 older adults (baseline mean age 74 years) from Cuba, Dominican Republic, Mexico, Peru, Puerto Rico, and Venezuela. The baseline examinations were done in 2003-2007 and the follow-up examinations 4 years later. Semi-structured psychiatric interviews gave information about ICD-10 depression and sub-syndromal depression (i.e., ≥4 depressive symptoms) at baseline. Information on dementia were collected at the follow-up examination. Competing risk models analyzed the associations between depression and incidence of dementia and the final model were adjusted for age, sex, education, stroke, and diabetes. Separate analyses were conducted for each site and then meta-analyzed by means of fixed effect models.

**Results:**

At baseline, the prevalence of depression was 26.0% (*n* = 2,980): 5.4% had ICD-10 depression and 20.6% sub-syndromal depression. During the follow-up period, 9.3% (*n* = 862) developed dementia and 14.3% (*n* = 1,329) deceased. In the pooled analyses, both ICD-10 depression (adjusted sub-hazard ratio (sHR) 1.63, 95% confidence interval (CI) 1.26–2.11) and sub-syndromal depression (adjusted sHR 1.28, 95% CI: 1.09–1.51) were associated with increased incidence of dementia. The Higging I^2^ tests showed a moderate heterogeneity across the study sites.

**Conclusion:**

Our findings suggest that late-life depression is associated with the incidence of dementia in LMICs in Latin America, which support results from earlier studies conducted in high-income countries.

## INTRODUCTION

Demographic aging is a worldwide phenomenon and number of people with dementia are increasing rapidly [1]. In low- and middle-income countries (LMICs), dementia cases are expected to more than triple by 2050 [1]. Latin America have a high prevalence of dementia (standardized prevalence = 8.3), compared to, for example, North America (5.7), Asia (5.6–7.7), Africa (4.6), and Europe (4.7–6.7) [2]. In addition, depression is a common condition in late-life [3, 4], and population-based studies report that as many as one third of older adults in LMICs and Latin America could be affected by depressive syndromes [4, 5].

A growing body of evidence suggests that late-life depression is related to onset of dementia. Several epidemiological studies have observed this relationship and meta-analyses have repeatedly reported a two-fold higher risk for dementia in people with depression [6–9]. The characteristics of this relationship are not yet sufficiently understood and some studies report contradictory results, i.e., that depression has no effect of the trajectory of dementia [10, 11]. While progress has been made, a glaring omission in the literature shows that almost all prior studies on this topic have been conducted in high-income countries. According to the latest systematic review, only one study was carried out in a middle-income country, i.e., in China [9].

The association between depression and onset of dementia can be explained in several ways. Depression can be a trigger for damaging neuropathological processes in the brain, e.g., through alterations in the nervous, vascular, and inflammatory systems [12]. Episodes of severe depression have earlier been associated with pathological changes in specific regions in the brain, e.g., in the prefrontal cortex, [13] in the hippocampus area [14], and in sub-cortical white matter [15]. Symptoms of depression may also be a psychological reaction to a cognitive and functional decline, or reflect a prodromal state of dementia.

While existing studies on the association between depression and dementia show inconsistency and mainly have been carried out in high-income countries [6–9], it is not known whether this relation can be found in other settings. The aim of this study was to examine the association between depression and incidence of dementia over a 4-year follow-up period, in a large population-based sample of older people living in six LMICs in Latin America.

## METHODS

### Setting and participants

The analyses originate from the 10/66 Dementia Research Group population-based survey, which include persons aged≥65 years living in geographically defined catchment areas in urban and rural sites in Latin America [1, 16]. Baseline examinations were carried out between 2003 and 2007 (depending on site) and included clinical interviews, physical examinations, and informant interviews, which generated information about, e.g., cognitive and mental disorders, physical health, socio-demographic factors, disability, and use of health services. Trained interviewers examined the participants’ in their own homes (2-3 hours) and all instruments were translated, back translated, and assessed for acceptance and conceptual equivalence [17]. The follow-up examinations were conducted 3 to 5 years after baseline and were essentially a repeat of the first examination. The 10/66 study has earlier been described in details [1, 16].

**Fig.1:**
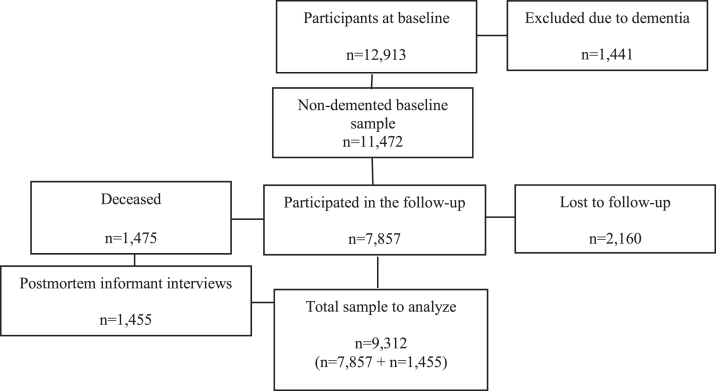
Flow-chart of participants at baseline and follow-up.

Persons with dementia at baseline (*n* = 1,441) were removed from all analyses. Of the remaining 11,472 persons, 7,857 participated in the follow-up examination (2,160 were lost to follow up and 1,475 had deceased). Postmortem informant interviews were done in the majority of people who were deceased (*n* = 1,455, 99%), using the World Health Organization’s “*Standard Verbal Autopsy Questionnaire 3: Death of a Person Aged 15 Years and Above*”, as described elsewhere [18]. The final analyses included 9,312 participants from Cuba (*n* = 2,339), Dominican Republic (*n* = 1,441), Peru (*n* = 1,328), Venezuela (*n* = 1,353), Mexico (*n* = 1,521), and Puerto Rico (*n* = 1,374). (See flow-chart in Fig. 1.) All subjects provided informed consent to participate, either by themselves or through their next-of-kin. The data collection and study procedure were approved by the local ethics committees and by the King’s College; London Research Ethics Committee.

### Diagnosis of depression

Two definitions of depression were used: (I) *ICD-10 depression* (code F32) [19] and (II) *sub-syndromal depression* (≥4 symptoms in the EURO-D scale, but not fulfilling the ICD-10 criteria). Both ICD-10 and EURO-D items were drawn from the semi-structured Geriatric Mental State (GMS) interview, which assess symptoms of depression during the last month [20]. The GMS interview is a widely used mental-health assessment for older people. The EURO-D symptom scale include 12 depressive symptoms, i.e., depressed mood, pessimism, wishing death, guilt, sleep, interest, irritability, appetite, fatigue, concentration, enjoyment, and tearfulness. Each item score 1 (present symptom) or 0 (no present symptom) [21]. The scale was originally developed to compare symptoms of late-life depression across European countries in the EURODEP Concerted Action Programme [22]. A computerized diagnostic algorithm, the AGECAT (Automated Geriatric Examination for Computer Assisted Taxonomy) grouped symptoms to identify diagnoses, according to, e.g., the ICD-10 criteria [19, 20, 23, 24]. The reliability and validity of the GMS/AGECAT algorithm has been investigated in several previous studies [4, 25].

### Diagnosis of dementia

Dementia was defined through an algorithm authorized by the 10/66 Dementia Research Group [26]. The algorithm has been calibrated and validated in a large cross-cultural pilot study and is described in details elsewhere [17, 26]. The diagnosis of dementia was allocated to individuals scoring above a cut-off point of predicted probability for dementia, calculated with coefficients derived from a logistic regression equation, and based on the combined information from: 1) the GMS interview [21, 24], 2) cognitive tests, i.e., the Community Screening Instrument for Dementia (CSI‘D’) [27] and a verbal fluency test [28], and 3) informant reports of cognitive and functional decline (from the CSI‘D’ instrument) [29]. For individuals who died between baseline and follow-up, a postmortem informant interview was used to assess whether there was evidence of cognitive and functional decline [26]. Participants who were suspected to have died with dementia were coded as ‘cases of dementia’.

### Potential confounders

A number of socio-demographic and health characteristics were added to the analyses. They were all selected on the basis that they either were assumed to have a potential confounding effect on the association between depression and dementia, or were used in previous studies researching this association. The potential confounders included age (continuous in year), gender (female/male), educational level (none, not completed primary, completed primary, completed secondary and tertiary education), stroke (“*Have you ever been told by a doctor that you had a stroke*?”; coded yes if there was a clear history of sudden onset of unilateral paralysis, loss of speech, or blindness lasting for more than 24 hours) [30], and diabetes (“*Have you ever been told by a doctor you had diabetes*?” coded yes/no) [30].

### Statistical analyses

First, we analyzed the socio-demographic and health characteristics for each study site and for the total sample. Second, we calculated person-years from the date of baseline examination to 1) the date of death in persons deceased without dementia, 2) the date of follow-up interview in survivors free from dementia, or 3) the date of dementia onset (i.e., the midpoint between the baseline examination and the follow-up examination or postmortem interview). Third, we tested associations between ICD-10 depression and dementia, and sub-syndromal depression and dementia, acknowledging the possibility of a dementia-free death (competing event). The reference group were participants without depression. Sub-hazard ratios (sHR) were derived from Fine and Gray’s proportional hazard models [31]. To test the proportionality of sub-hazards, we included time interactions on all the co-variates as a way of testing the proportional sub-hazards assumption for each. We found that no indication that proportionality assumption had been violated.

Competing risk models were run separately in each site and then pooled together by fixed effect meta-analysis, using inverse-variance weighting together with an estimation of heterogeneity using the Higgins I2. Higgins I2 was computed to estimate the proportion of between-site variability in the estimates accounted for by heterogeneity as opposed to sampling error (up to 40% heterogeneity is conventionally considered negligible, while up to 60% reflects moderate heterogeneity). The 1st model was unadjusted, the 2nd model adjusted for age, gender, and education level, and the 3rd model adjusted for age, gender, education level, stroke, and diabetes.

The statistical significance was defined as a *p*-value <.05 (two-tailed) in all analyses.

Finally, three additional sensitivity analyses where conducted: 1) a competing risk regression model was used to repeat the analyses and categorizing everyone who died as “deceased without dementia”, i.e., not using the postmortem informant information about dementia at time of death (Supplementary Table 1), 2) we repeated the analyses using Cox regression models instead of competing risk models (Supplementary Table 2), and finally 3) we repeated the meta-analyses to pool countries together by removing one country at the time, i.e., leave-one-out validation. This was done to assess the impact that each individual site had on the pooled estimates and the robustness of the findings. All data analysis was performed using Stata 13 (StataCorp, College Station, TX, USA).

## RESULTS

The characteristics of the study participants (*n* = 11,472, 64% women) are presented in Table 1. At baseline (mean age = 74.0±6.7), the prevalence of ICD-10 depression was 5%, whereas 21% met the criteria for sub-syndromal depression. Depression and sub-syndromal depression was more common in women than in men (31% versus 18%) and varied between study sites, from 16% in Puerto Rico to 37% in the Dominican Republic. During the 4 years of follow-up, 862 of the participants developed dementia, 736 were detected at the examination, and 126 in the postmortem informant interviews. The incidence of dementia was slightly more common in women than in men (10% versus 8%) and varied from 6% in Peru to 12% in Dominican Republic and Venezuela. One thousand fifty-five participants deceased without a diagnosis of dementia.

**Table 1:** Sociodemographic characteristics of the sample

	Cuba	Dominican Republic	Peru	Venezuela	Mexico	Puerto Rico	Across centers
Number of participants at risk, n	2517	1769	1767	1820	1823	1776	**11472**
Median follow-up to outcome, y (IQR)	4.1 (3.4–5.0)	5.0 (3.3–5.1)	3.0 (2.5–3.7)	4.2 (3.9–4.7)	3.0 (2.9–3.1)	4.3 (3.7–4.7)	**3.8 (2.9–4.7)**
Mean age at baseline, y (SD)	74.4 (6.6)	74.5 (7.1)	74.2 (6.9)	72.0 (6.4)	73.6 (6.3)	75.4 (6.8)	**74.0 (6.8)**
Females, n (%)	1628 (64.7)	1156 (65.4)	1073 (60.7)	1150 (63.2)	1144 (62.8)	1190 (67.3)	**7341 (64.0)**
Prevalence baseline depression, n (%)
ICD-10 depressive episode	118 (4.7)	221 (12.5)	86 (4.9)	84 (4.6)	73 (4.0)	37 (2.0)	**619 (5.4)**
Sub-syndromal depression	455 (18.1)	417(23.6)	399 (22.6)	421 (23.1)	424 (23.3)	253 (14.3)	**2369 (20.6)**
Any depression	573 (22.8)	638 (36.1)	485 (27.5)	505 (27.7)	497 (27.3)	290 (16.3)	**2988 (26.0)**
Education level, n (%)
None	54 (2.1)	315 (17.9)	103 (5.9)	133 (7.4)	461 (25.3)	47 (2.6)	**1113 (9.7)**
Some	522 (20.8)	917 (52.0)	212 (12.1)	408 (22.6)	802 (44.0)	316 (17.8)	**3177 (27.8)**
Completed primary	829 (33.0)	338 (19.2)	654 (37.2)	913 (50.5)	337 (18.5)	358 (20.2)	**3429 (30.0)**
Completed secondary	661 (26.3)	126 (7.1)	486 (27.7)	262 (14.5)	117 (6.4)	663 (37.2)	**2315 (20.3)**
Tertiary (college)	446 (17.5)	66 (3.7)	301 (17.1)	92 (5.1)	104 (5.7)	385 (21.7)	**1394 (12.2)**
Stroke, n (%)	158 (6.3)	118 (6.7)	106 (6.0)	111 (6.1)	120 (6.6)	124 (7.6)	**737 (6.5)**
Diabetes, n (%)	466 (18.6)	253 (14.3)	158 (9.0)	283 (15.6)	398 (21.8)	569 (32.2)	**2127 (18.6)**
Status at follow up
Interviewed, n (%)	1851 (73.5)	1071 (60.4)	1214 (68.7)	1192 (65.5)	1355 (74.3)	1174 (66.5)	**7857 68.5)**
Deceased, n (%)	449 (17.8)	370 (20.9)	109 (6.2)	161 (8.9)	166 (9.1)	200 (11.3)	**1455 (12.7)**
Lost, n (%)	217 (8.6)	328 (18.5)	444 (25.1)	467 (25.7)	302 (16.6)	402 (22.7)	**2160 (18.8)**
Outcome
Censored, n (%)	1681 (73.1)	953 (66.1)	1145 (86.6)	1057 (78.1)	1234 (81.1)	1051 (76.5)	**7121 (76.5)**
Incident dementia, n (%)	182 (7.9)	165 (11.4)	77 (5.8)	155 (11.5)	130 (8.6)	153 (11.1)	**862 (9.3)**
Competing risk, n (%)	437 (19.0)	323 (22.4)	101 (7.6)	141 (10.4)	157 (10.3)	170 (12.4)	**1329 (14.3)**

IQR, interquartile range

In the pooled meta-analysis, people with ICD-10 depression had an 85% increased risk of developing dementia during follow-up period (sHR 1.85, 95% CI 1.44–2.37, I^2^ 45.9%), compared to people without depression (Table 2). The association remained after adjusting for potential confounders (pooled fully adjusted sHR 1.63, 95% CI 1.26–2.11, I^2^ 40.9%). The hazard estimates varied between the study sites. Cuba (sHR 2.53, 95% CI 1.58–4.07) and Venezuela (sHR 2.84, 95% CI 1.62–4.97) had the highest sHR, while Dominican Republic (sHR 1.18, 95% CI 0.75–1.85) and Puerto Rico (sHR 0.76, 95% CI 0.18–3.20) had the lowest observed estimations. Sub-syndromal depression was associated with a 37% increased risk of developing dementia (95% CI 1.17–1.60, I^2^ 53.7%) in the pooled meta-analysis (Table 3).

**Table 2:** Sub-hazard ratios for incident dementia in persons with ICD-10 depressive episode at baseline

	Unadjusted sHR (95% CI)^a^		Model 2 sHR (95% CI)^a^		Model 3 sHR (95% CI)^a^
Cuba	2.53 (1.58–4.07)		2.45 (1.50–4.01)		2.48 (1.52–4.06)
Dominican Republic	1.18 (0.75–1.85)		1.00 (0.62–1.06)		1.01 (0.62–1.62)
Peru	1.71 (0.74–3.96)		1.43 (0.64–3.22)		1.39 (0.61–3.19)
Venezuela	2.84 (1.62–4.97)		2.53 (1.40–4.59)		2.12 (1.16–3.87)
Mexico	1.82 (0.87–3.81)		1.79 (0.85–3.76)		1.82 (0.87–3.82)
Puerto Rico	0.76 (0.18–3.20)		0.87 (0.21–3.68)		0.81 (0.19–3.48)
		I^2^		I^2^		I^2^
**Pooled**	**1.85 (1.44–2.37)**	**45.9%**	**1.38 (1.12–1.68)**	**68.1%**	**1.63 (1.26–2.11)**	**40.9%**

Competing risk models presented using sub-hazard ratios (sHR) and 95% confidence interval (CI). I^2^, Higgins I^2^.; Model 1: unadjusted, Model 2: adjusted for age, gender, and education level, and Model 3: adjusted for age, gender, education level, stroke, and diabetes.; ^a^People with no depression were used as the reference group.

**Table 3:** Sub-hazard ratios for incident dementia in people with sub-syndromal depression at baseline

	Unadjusted sHR (95% CI)^a^		Model 2 sHR (95% CI)^a^		Model 3 sHR (95% CI)^a^
Cuba	0.98 (0.66–1.45)		0.89 (0.59–1.33)		0.88 (0.59–1.32)
Dominican Republic	1.21 (0.85–1.72)		1.11 (0.78–1.57)		1.10 (0.77–1.56)
Peru	0.99 (0.57–1.71)		0.82 (0.46–1.46)		0.82 (0.47–1.46)
Venezuela	2.10 (1.50–2.95)		2.22 (1.56–3.14)		2.13 (1.50–3.03)
Mexico	1.41 (0.96–2.05)		1.38 (0.94–2.01)		1.37 (0.93–2.02)
Puerto Rico	1.43 (0.96–2.14)		1.47 (0.97–2.22)		1.38 (0.90–2.12)
		I^2^		I^2^		I^2^
**Pooled**	**1.37 (1.17–1.60)**	**53.7%**	**1.32 (1.12–1.55)**	**68.7%**	**1.28 (1.09–1.51)**	**66.0%**

Competing risk models presented using sub-hazard ratios (sHR) and 95% confidence interval (CI). I2, Higgins I2.; Model 1: unadjusted, Model 2: adjusted for age, gender, and education level, and Model 3: adjusted for age, gender, education level, stroke, and diabetes.; ^a^people with no depression were used as the reference group.

When the analyses where repeated by categorizing everyone who died as ‘deceased without dementia’, i.e., not using the postmortem informant information about suspected dementia at time of death, the risk estimation of dementia where similar both for ICD-10 depression (pooled fully adjusted sHR 1.51) and sub-syndromal depression (pooled fully adjusted sHR 1.33) (Supplementary Table 1). Also the Cox regression models showed similar estimates as the competing risk models, both according to ICD-10 depression (pooled fully adjusted sHR 1.74) and sub-syndromal depression (pooled fully adjusted sHR 1.32) (Supplementary Table 2).

When repeated the meta-analyses and removing one study at the time, i.e., leave-one-out validation test, in Table 2, the pooled estimates did not change and their 95% CI did not overlap. Removing Venezuela from Model 3 resulted in a pooled estimate of sHR 1.12 (95% CI 0.93–1.34). Removing other countries did not affect the results.

## DISCUSSION

In a large population-based sample of older adults in six defined regions in Latin America, we found that depression was associated with a higher incidence of dementia over a 4-year follow-up period. The strongest associations were observed in people with ICD-10 depression but also in people with sub-syndromal depression (≥4 symptoms in the EURO-D, no confirmed ICD-10 depression) had higher risk of developing dementia. The study sites differed both according to prevalence of depression (e.g., ICD-10 depression ranged from 13% to 25%) and according to the hazard estimates (adjusted sHRs ranged between 0.81 and 2.48 for ICD-10 depression).

To the best of our knowledge, the present 10/66-study is the first longitudinal study examining the association between depressive symptoms and incidence of dementia in LMICs and Latin America. Only a few cross-sectional studies have previously reported on the associations between depression and dementia in older people living in LMIC populations [32–34]. The findings are in line with previous published meta-analyses conducted in high-income populations [7–9]. A report from Alzheimer’s Disease International (ADI) presented pooled data from 32 studies (*n* = 62,598, baseline mean age ranged 70-89 years, median follow-up 5 years) and showed that depression doubled the risk of developing dementia [6].

The prevalence of cognitive and affective disorders differs between countries and cultures around world. Today, the majority of people with dementia lives in LMICs [26], and it is estimated that these countries will see the most rapid increase of dementia in the following decades [1]. In addition, depression is a common health problem in late-life [3] and population-based studies in LMICs and Latin America report that up to one third of older adults can be affected by depressive symptomatology [4, 5]. The incidence of depression has, however, considerable variations both in Latin America populations [4] and in other parts of the world [35], most likely as a consequence of cultural, demographic, and socio-economic factors. This high variation may also be due to methodological issues and differences in evaluation of symptoms. In the 10/66-study, every effort was made to ensure a conceptual equivalence of all interview questionnaires’ items. The research teams underwent substantial training to ensure a consistent approach in the administration of assessments across different cultural settings in accordance with manualized standard operational procedures. The variation in prevalence of depression between the study sites is therefore unlikely to be the result of measurement errors.

The underlying mechanisms by which depression is associated with dementia in older adults are complex and may have several explanations. It remains unclear whether depression itself initiates or worsen the trajectory of dementia, or if depression is an early sign of incipient dementia. The most prominent biological mechanisms that may link depression to dementia are likely to be the results of shared nervous, [36, 37] vascular, [38] and inflammatory [39] pathways. For example, depression increases the activity of the hypothalamic-pituitary-adrenal (HPA) axis and thus the levels of glucocorticoid hormones in the brain, which in turn have been associated with hippocampus atrophy and memory problems [36]. Depression is also associated with dysregulation in the nervous system, e.g., low serotonin and noradrenaline levels in brain, which have been related to impaired cognitive function [37]. Another explanation could be that depression is associated with prolonged low-grade inflammation that in the long-term may have a damaging effect on neurons and brain blood vessels [40].

It is well known that the neurodegenerative processes in the brain often starts many years before a person fulfil the diagnostic criteria for dementia [41]. Early neurodegenerative changes in the brain may increase the vulnerability to depression and the association between depression and incipient dementia can thus reflect a prodromal state of dementia. In our case, 4 years of follow-up is a relative short period of monitoring, which made it difficult to fully establish the direction of the association, e.g., that the depression itself causes changes that lead to dementia. Cognitive and physical function due to incipient dementia process may also have impact on the mental wellbeing and result in psychiatric symptoms, such as low mood and anxiety [42].

Cognitive symptoms are common in the context of depression and mood symptoms frequently accompany cognitive disorders. Several symptoms of depression, e.g., concentration problems, sleeping problems, agitation, fatigue, and lack of appetite, are also common in people with dementia, and partly used as diagnostic criteria [19, 22]. Different types of underlying neuropathology may thus appear as shared clinical symptomatology. The 10/66-study has however no biological measurements, such as magnetic resonance imaging or cerebrospinal fluid markers, to identify specific brain pathologies.

It can be a challenge for clinicians assessing and treating older patients, with co-morbid cognitive and affective symptoms. Studies on psychopharmacology drugs show inconsequent results, and antidepressant treatment has been associated with both increased [43] and reduced [44] risk of dementia.

### Strength and limitations

The strengths of this study include a large population-based sample in LMICs, a longitudinal study design, face-to-face interviews, and a systematic data collection with identical standardized protocols. Assessment of depression was done by a validated and structured interview that was designed specifically for these older populations, which helped in comparing results across countries using a standardized method. Some methodological issues need however to be considered. First, lack of data on sub-types of dementia, e.g., Alzheimer’s disease or vascular dementia, limit our understanding of this association in respect to underlying neurobiological processes. Second, we have no information about the time of onset of depression. It is possible that the association between depression and dementia may be affected by the duration of depression, the numbers of episodes, and the type of treatment received. Third, no information was available on psychopharmacological treatment, i.e., anti-depressant medication or psychological intervention, which can influence the association between depression and dementia. Fourth, some residual confounding factors which were not measured could still exist. For example, other physical and mental health conditions, and lifestyle factors such as, e.g., smoking, alcohol, and stress. Fifth, in people who deceased between baseline and follow-up, information on dementia was assessed using a postmortem informant interview, which may have lower validation than the standardized diagnostically processes from the examinations. The same approach has, however, been used in several previous 10/66 studies [45, 46], and a sensitivity analysis, which did not include the postmortem information did not show any major difference in regards to the findings. Sixth, cumulative attrition is a problem in long follow-up studies and the participation in the follow-up examination may thus be healthier and have less dementia than the general population. Finally, people with mild cognitive impairment were not excluded from baseline. This could potentially have affected the results of the study, as pathological changes in the brain may have already started occurring in this group of participants.

### Conclusion and implications

Our study found that depression was associated with incidence of dementia over a 4-year follow-up period, even though variation was found across countries. Further research needs to explored more regarding this variance, to understand whether contextual factors may explain these differences. For clinicians, this study strengthens the evidence supporting the importance of a cognitive examination of older people who present with depressive symptoms.

## Supplementary Material

Supplementary Material

## References

[1] Prince M , Ferri CP , Acosta D , Albanese E , Arizaga R , Dewey M , Gavrilova SI , Guerra M , Huang Y , Jacob KS , Krishnamoorthy ES , McKeigue P , Rodriguez JL , Salas A , Sosa AL , Sousa RM , Stewart R , Uwakwe R (2007) The protocols for the 10/66 dementia research group population-based research programme. BMC Public Health 7, 165. 17659078 10.1186/1471-2458-7-165 PMC1965476

[2] Prince MJ (2015) World Alzheimer Report 2015: The global impact of dementia: An analysis of prevalence, incidence, cost and trends. Alzheimer’s Disease International (ADI), London.

[3] Panza F , Frisardi V , Capurso C , D’Introno A , Colacicco AM , Imbimbo BP , Santamato A , Vendemiale G , Seripa D , Pilotto A , Capurso A , Solfrizzi V (2010) Late-life depression, mild cognitive impairment, and dementia: Possible continuum? Am J Geriatr Psychiatry 18, 98–116. 20104067 10.1097/JGP.0b013e3181b0fa13

[4] Guerra M , Prina AM , Ferri CP , Acosta D , Gallardo S , Huang Y , Jacob KS , Jimenez-Velazquez IZ , Llibre Rodriguez JJ , Liu Z , Salas A , Sosa AL , Williams JD , Uwakwe R , Prince M (2016) A comparative cross-cultural study of the prevalence of late life depression in low and middle income countries. J Affect Disord 190, 362–368. 26544620 10.1016/j.jad.2015.09.004 PMC4679114

[5] Guerra M , Ferri CP , Sosa AL , Salas A , Gaona C , Gonzales V , de la Torre GR , Prince M (2009) Late-life depression in Peru, Mexico and Venezuela: The 10/66 population-based study. Br J Psychiatry 195, 510–515. 19949200 10.1192/bjp.bp.109.064055 PMC2915389

[6] Prince M , Albanese E , Guerchet M , Prina AM (2014) Dementia and risk reduction: An analysis of protective and modifiable factors. Alzheimer’s Disease International (ADI), London.

[7] Diniz BS , Butters MA , Albert SM , Dew MA , Reynolds CF 3rd (2013) Late-life depression and risk of vascular dementia and Alzheimer’s disease: Systematic review and meta-analysis of community-based cohort studies. Br J Psychiatry 202, 329–335. 23637108 10.1192/bjp.bp.112.118307 PMC3640214

[8] Ownby RL , Crocco E , Acevedo A , John V , Loewenstein D (2006) Depression and risk for Alzheimer disease: Systematic review, meta-analysis, and metaregression analysis. Arch Gen Psychiatry 63, 530–538. 16651510 10.1001/archpsyc.63.5.530 PMC3530614

[9] Cherbuin N , Kim S , Anstey KJ (2015) Dementia risk estimates associated with measures of depression: A systematic review and meta-analysis. BMJ Open 5, e008853. 10.1136/bmjopen-2015-008853 PMC4691713 26692556

[10] Geerlings MI , den Heijer T , Koudstaal PJ , Hofman A , Breteler MM (2008) History of depression, depressive symptoms, and medial temporal lobe atrophy and the risk of Alzheimer disease. Neurology 70, 1258–1264. 18391157 10.1212/01.wnl.0000308937.30473.d1

[11] Palsson S , Aevarsson O , Skoog I (1999) Depression, cerebral atrophy, cognitive performance and incidence of dementia. Population study of 85-year-olds. Br J Psychiatry 174, 249–253. 10448451 10.1192/bjp.174.3.249

[12] Byers AL , Yaffe K (2011) Depression and risk of developing dementia. Nat Rev Neurol 7, 323–331. 21537355 10.1038/nrneurol.2011.60 PMC3327554

[13] Almeida OP , Burton EJ , Ferrier N , McKeith IG , O’Brien JT (2003) Depression with late onset is associated with right frontal lobe atrophy. Psychol Med 33, 675–681. 12785469 10.1017/s003329170300758x

[14] Lorenzetti V , Allen NB , Fornito A , Yucel M (2009) Structural brain abnormalities in major depressive disorder: A selective review of recent MRI studies. J Affect Disord 117, 1–17. 19237202 10.1016/j.jad.2008.11.021

[15] Gudmundsson P , Olesen PJ , Simoni M , Pantoni L , Ostling S , Kern S , Guo X , Skoog I (2015) White matter lesions and temporal lobe atrophy related to incidence of both dementia and major depression in 70-year-olds followed over 10 years. Eur J Neurol 22, 781–788, e749-750. 25598324 10.1111/ene.12651

[16] Prina AM , Acosta D , Acostas I , Guerra M , Huang Y , Jotheeswaran AT , Jimenez-Velazquez IZ , Liu Z , Llibre Rodriguez JJ , Salas A , Sosa AL , Williams JD , Prince M (2016) Cohort Profile: The 10/66 study. Int J Epidemiol 46, 406–406i. 10.1093/ije/dyw056 PMC5837706 27154633

[17] Prince M , Acosta D , Chiu H , Scazufca M , Varghese M (2003) Dementia diagnosis in developing countries: A cross-cultural validation study. Lancet 361, 909–917. 12648969 10.1016/S0140-6736(03)12772-9

[18] Ferri CP , Acosta D , Guerra M , Huang Y , Llibre-Rodriguez JJ , Salas A , Sosa AL , Williams JD , Gaona C , Liu Z , Noriega-Fernandez L , Jotheeswaran AT , Prince MJ (2012) Socioeconomic factors and all cause and cause-specific mortality among older people in Latin America, India, and China: A population-based cohort study. PLoS Med 9, e1001179. 22389633 10.1371/journal.pmed.1001179 PMC3289608

[19] World Health Organization (1992) The ICD–10 Classification of Mental and Behavioral Disorders. Diagnostic Criteria for Research.

[20] Copeland JR , Prince M , Wilson KC , Dewey ME , Payne J , Gurland B (2002) The Geriatric Mental State Examination in the 21st century. Int J Geriatr Psychiatry 17, 729–732. 12211122 10.1002/gps.667

[21] Copeland JR , Dewey ME , Griffiths-Jones HM (1986) A computerized psychiatric diagnostic system and case nomenclature for elderly subjects: GMS and AGECAT. Psychol Med 16, 89–99. 3515380 10.1017/s0033291700057779

[22] Prince MJ , Reischies F , Beekman AT , Fuhrer R , Jonker C , Kivela SL , Lawlor BA , Lobo A , Magnusson H , Fichter M , van Oyen H , Roelands M , Skoog I , Turrina C , Copeland JR (1999) Development of the EURO-D scale–a European, Union initiative to compare symptoms of depression in 14 European centres. Br J Psychiatry 174, 330–338. 10533552 10.1192/bjp.174.4.330

[23] Copeland JR , Dewey ME , Henderson AS , Kay DW , Neal CD , Harrison MA , McWilliam C , Forshaw D , Shiwach R (1988) The Geriatric Mental State (GMS) used in the community: Replication studies of the computerized diagnosis AGECAT. Psychol Med 18, 219–223. 3283808 10.1017/s003329170000204x

[24] Copeland JR , Kelleher MJ , Kellett JM , Gourlay AJ , Gurland BJ , Fleiss JL , Sharpe L (1976) A semi-structured clinical interview for the assessment of diagnosis and mental state in the elderly: The Geriatric Mental State Schedule. I. Development and reliability. Psychol Med 6, 439–449. 996204 10.1017/s0033291700015889

[25] Collighan G , Macdonald A , Herzberg J , Philpot M , Lindesay J (1993) An evaluation of the multidisciplinary approach to psychiatric diagnosis in elderly people. BMJ 306, 821–824. 8490373 10.1136/bmj.306.6881.821 PMC1677287

[26] Prince M , Acosta D , Ferri CP , Guerra M , Huang Y , Llibre Rodriguez JJ , Salas A , Sosa AL , Williams JD , Dewey ME , Acosta I , Jotheeswaran AT , Liu Z (2012) Dementia incidence and mortality in middle-income countries, and associations with indicators of cognitive reserve: A 10/66 Dementia Research Group population-based cohort study. Lancet 380, 50–58. 22626851 10.1016/S0140-6736(12)60399-7 PMC3525981

[27] Hall KS , Hendrie HC , Brittain HM , Norton JA , Rodgers DD , Prince CS , Pillay N , Blue AW , Kaufert JN , Nath A , Shelton P , Postl BD , Osuntokun BO (1993) The development of a dementia screening interview in 2 distinct languages. Int J Methods Psychiatr Res 3, 1–28.

[28] Ganguli M , Chandra V , Gilby JE , Ratcliff G , Sharma SD , Pandav R , Seaberg EC , Belle S (1996) Cognitive test performance in a community-based nondemented elderly sample in rural India: The Indo-U.S. Cross-National Dementia Epidemiology Study. Int Psychogeriatr 8, 507–524. 9147167 10.1017/s1041610296002852

[29] Hall KS , Gao S , Emsley CL , Ogunniyi AO , Morgan O , Hendrie HC (2000) Community screening interview for dementia (CSI ‘D’); performance in five disparate study sites. Int J Geriatr Psychiatry 15, 521–531. 10861918 10.1002/1099-1166(200006)15:6<521::aid-gps182>3.0.co;2-f

[30] Sousa RM , Ferri CP , Acosta D , Albanese E , Guerra M , Huang Y , Jacob KS , Jotheeswaran AT , Rodriguez JJ , Pichardo GR , Rodriguez MC , Salas A , Sosa AL , Williams J , Zuniga T , Prince M (2009) Contribution of chronic diseases to disability in elderly people in countries with low and middle incomes: A 10/66 Dementia Research Group population-based survey. Lancet 374, 1821–1830. 19944863 10.1016/S0140-6736(09)61829-8 PMC2854331

[31] Fine JP , Gray RJ (1999) A proportional hazards model for the subdistribution of a competing risk. J Am Stat Assoc 94, 496–509.

[32] Tsolaki M , Gkioka M , Verykouki E , Galoutzi N , Kavalou E , Pattakou-Parasyri V (2017) Prevalence of dementia, depression, and mild cognitive impairment in a rural area of the island of Crete, Greece. Am J Alzheimers Dis Other Demen 32, 252–264. 28468554 10.1177/1533317517698789 PMC10852845

[33] Li XL , Hu N , Tan MS , Yu JT , Tan L (2014) Behavioral and psychological symptoms in Alzheimer’s disease. Biomed Res Int 2014, 927804. 25133184 10.1155/2014/927804 PMC4123596

[34] Andreasen P , Lonnroos E , von Euler-Chelpin MC (2014) Prevalence of depression among older adults with dementia living in low- and middle-income countries: A cross-sectional study. Eur J Public Health 24, 40–44. 10.1093/eurpub/ckt014 23417621

[35] Castro-Costa E , Dewey M , Stewart R , Banerjee S , Huppert F , Mendonca-Lima C , Bula C , Reisches F , Wancata J , Ritchie K , Tsolaki M , Mateos R , Prince M (2007) Prevalence of depressive symptoms and syndromes in later life in ten European countries: The SHARE study. Br J Psychiatry 191, 393–401. 17978318 10.1192/bjp.bp.107.036772

[36] Belvederi Murri M , Pariante C , Mondelli V , Masotti M , Atti AR , Mellacqua Z , Antonioli M , Ghio L , Menchetti M , Zanetidou S , Innamorati M , Amore M (2014) HPA axis and aging in depression: Systematic review and meta-analysis. Psychoneuroendocrinology 41, 46–62. 24495607 10.1016/j.psyneuen.2013.12.004

[37] Meltzer CC , Smith G , DeKosky ST , Pollock BG , Mathis CA , Moore RY , Kupfer DJ , Reynolds CF 3rd (1998) Serotonin in aging, late-life depression, and Alzheimer’s disease: The emerging role of functional imaging. Neuropsychopharmacology 18, 407–430. 9571651 10.1016/S0893-133X(97)00194-2

[38] Aarsland D , Sardahaee FS , Anderssen S , Ballard C , Alzheimer’s Society Systematic Review Group (2010) Is physical activity a potential preventive factor for vascular dementia? A systematic review. Aging Ment Health 14, 386–395. 20455113 10.1080/13607860903586136

[39] Hayley S , Audet MC , Anisman H (2016) Inflammation and the microbiome: Implications for depressive disorders. Curr Opin Pharmacol 29, 42–46. 27327647 10.1016/j.coph.2016.06.001

[40] Leonard BE (2018) Inflammation and depression: A causal or coincidental link to the pathophysiology? Acta Neuropsychiatr 30, 1–16. 10.1017/neu.2016.69 28112061

[41] Ringman JM , Younkin SG , Pratico D , Seltzer W , Cole GM , Geschwind DH , Rodriguez-Agudelo Y , Schaffer B , Fein J , Sokolow S , Rosario ER , Gylys KH , Varpetian A , Medina LD , Cummings JL (2008) Biochemical markers in persons with preclinical familial Alzheimer disease. Neurology 71, 85–92. 18509095 10.1212/01.wnl.0000303973.71803.81 PMC12435649

[42] Borsje P , Wetzels RB , Lucassen PL , Pot AM , Koopmans RT (2015) The course of neuropsychiatric symptoms in community-dwelling patients with dementia: A systematic review. Int Psychogeriatr 27, 385–405. 25403309 10.1017/S1041610214002282

[43] Almeida OP , Hankey GJ , Yeap BB , Golledge J , Flicker L (2017) Depression as a modifiable factor to decrease the risk of dementia. Transl Psychiatry 7, e1117. 28463236 10.1038/tp.2017.90 PMC5534958

[44] Kessing LV , Forman JL , Andersen PK (2011) Do continued antidepressants protect against dementia in patients with severe depressive disorder? Int Clin Psychopharmacol 26, 316–322. 21876440 10.1097/YIC.0b013e32834ace0f

[45] Prince MJ , Acosta D , Guerra M , Huang Y , Jimenez-Velazquez IZ , Llibre Rodriguez JJ , Salas A , Sosa AL , Dewey ME , Guerchet MM , Liu Z , Llibre Guerra JJ , Prina AM (2018) Leg length, skull circumference, and the incidence of dementia in Latin America and China: A 10/66 population-based cohort study. PLoS One 13, e0195133. 29649337 10.1371/journal.pone.0195133 PMC5896923

[46] Prince MJ , Acosta D , Guerra M , Huang Y , Jimenez-Velazquez IZ , Llibre Rodriguez JJ , Salas A , Sosa AL , Chua KC , Dewey ME , Liu Z , Mayston R , Valhuerdi A (2018) Reproductive period, endogenous estrogen exposure and dementia incidence among women in Latin America and China; A 10/66 population-based cohort study. PLoS One 13, e0192889. 29489847 10.1371/journal.pone.0192889 PMC5831083

