# Supplementary material for: Associations between Depression, Depressive Symptoms, and Incidence of Dementia in Latin America: A 10/66 Dementia Research Group Study
Source: J Alzheimers Dis. 2019 May 21;69(2):433–41. doi: 10.3233/JAD-190148 (PMC6598112; doi:10.3233/JAD-190148)
Supplement: Supplementary Material [file jad-69-jad190148-s001.docx]

**Supplementary Material**

**Associations between Depression, Depressive Symptoms, and Incidence of Dementia in Latin America: A 10/66 Dementia Research Group Study**

**Supplementary Table 1**. Sub-hazard ratios for incident dementia, in people with depressive episode at baseline. Participants who died before the follow-up examination were considered as deceased (the competing risk) in the analyses, and the results of the verbal autopsies were not taken into consideration.

|  |  | **Unadjusted**  sHR (95% CI) ^a^ |  | **Model 2**  sHR (95% CI) ^a^ |  | **Model 3**  sHR (95% CI) ^a^ |  |
| --- | --- | --- | --- | --- | --- | --- | --- |
| Cuba | ICD-10 depression  Sub-syndromal depression | 2.08 (1.22-3.52)  1.00 (0.68-1.49) |  | 1.92 (1.11-3.33)  0.90 (0.60-1.36) |  | 1.95 (1.12-3.36)  0.89 (0.59-1.35) |  |
| Dominican Republic | ICD-10 depression  Sub-syndromal depression | 1.12 (0.64-1.97)  1.46 (0.98-2.18) |  | 0.92 (0.51-1.67)  1.27 (0.86-1.89) |  | 0.92 (0.50-1.68)  1.25 (0.84-1.86) |  |
| Peru | ICD-10 depression  Sub-syndromal depression | 1.60 (0.64-4.01)  1.05 (0.60-1.86) |  | 1.33 (0.55-3.19)  0.93 (0.51-1.66) |  | 1.28 (0.52-3.14)  0.93 (0.52-1.66) |  |
| Venezuela | ICD-10 depression  Sub-syndromal depression | 2.44 (1.30-4.59)  2.04 (1.42-2.93) |  | 2.07 (1.02-4.19)  2.11 (1.46-3.05) |  | 1.84 (0.89-3.82)  2.07 (1.42-3.00) |  |
| Mexico | ICD-10 depression  Sub-syndromal depression | 1.96 (0.93-4.10)  1.39 (0.94-2.07) |  | 1.93 (0.22-4.04)  1.37 (0.92-2.02) |  | 1.97 (0.94-4.11)  1.36 (0.91-2.03) |  |
| Puerto Rico | ICD-10 depression  Sub-syndromal depression | 0.99 (0.24-4.16)  1.55 (1.00-2.40) |  | 1.11 (0.27-4.64)  1.56 (1.00-2.45) |  | 1.05 (0.25-4.47)  1.50 (0.96-2.35) |  |
|  |  |  | **I^2^** |  | **I^2^** |  | **I^2^** |
| **Pooled** | ICD-10 depression  Sub-syndromal depression | **1.73 (1.31-2.29)**  **1.42 (1.20-1.68)** | **0%**  **39%** | **1.48 (1.09-2.02)**  **1.40 (1.18-1.66)** | **0%**  **48.7%** | **1.51 (1.12-2.02)**  **1.33 (1.12-1.59)** | **0%**  **53.9** |

Competing risk models presented using sub-hazard ratio (sHR) and 95% confidence interval (CI). I^2^, Higgins I^2^

Model 1: unadjusted, Model 2: adjusted for age, gender, and education level, and Model 3: adjusted for age, gender, education level, stroke, and diabetes

^a^ people with no depression were used as the reference group

**Supplementary Table 2.** Hazard ratios for incident dementia in people with ICD-depression and sub-syndromal depression, using Cox regression analyses.

|  |  | **Unadjusted**  HR (95% CI) ^a^ |  | **Model 2**  HR (95% CI) ^a^ |  | **Model 3**  HR (95% CI) ^a^ |  |
| --- | --- | --- | --- | --- | --- | --- | --- |
| Cuba | ICD-10 depression  Sub-syndromal depression | 2.57 (1.59-4.17)  1.02 (0.69-1.51) |  | 2.58 (1.58-4.20)  0.95 (0.64-1.42) |  | 2.55 (1.56-4.18)  0.93 (0.62-1.39) |  |
| Dominican Republic | ICD-10 depression  Sub-syndromal depression | 1.29 (0.82-2.04)  1.23 (0.87-1.75) |  | 1.14 (0.72-1.81)  1.12 (0.78-1.61) |  | 1.15 (0.72-1.82)  1.11 (0.78-1.61) |  |
| Peru | ICD-10 depression  Sub-syndromal depression | 1.84 (0.79-4.27)  0.99 (0.57-1.70) |  | 1.40 (0.60-3.26)  0.82 (0.47-1.44) |  | 1.34 (0.57-3.15)  0.82 (0.47-1.44) |  |
| Venezuela | ICD-10 depression  Sub-syndromal depression | 3.12 (1.77-4.49)  2.14 (1.52-3.00) |  | 3.21 (1.79-5.77)  2.38 (1.69-3.36) |  | 2.63 (1.41-4.92)  2.29 (1.61-3.24) |  |
| Mexico | ICD-10 depression  Sub-syndromal depression | 1.81 (0.88-3.75)  1.42 (0.97-2.07) |  | 1.80 (0.87-3.72)  1.38 (0.94-2.02) |  | 1.83 (0.88-3.80)  1.38 (0.94-2.02) |  |
| Puerto Rico | ICD-10 depression  Sub-syndromal depression | 0.79 (0.20-3.21)  1.40 (0.94-2.10) |  | 0.85 (0.21-3.45)  1.44 (0.96-2.17) |  | 0.76 (0.19-3.13)  1.36 (0.89-2.04) |  |
|  |  |  | **I^2^** |  | **I^2^** |  | **I^2^** |
| **Pooled** | ICD-10 depression  Sub-syndromal depression | **2.03 (1.60-2.57)**  **1.38 (1.18-1.62)** | **49.6%**  **52.6%** | **1.84 (1.43-2.37)**  **1.35 (1.15-1.59)** | **54.7% 71.6%** | **1.74 (1.35-2.25)**  **1.32 (1.12-1.55)** | **43.0%**  **69.1%** |

Cox regressions presented using by hazard ratios (HR) and 95% confidence interval (CI). I^2^, Higgins I^2^

Model 1: unadjusted, Model 2: adjusted for age, gender, and education level, and Model 3: adjusted for age, gender, education level, stroke, and diabetes

^a^ people with no depression were used as the reference group
